# A region of mumps virus nucleoprotein affects defective interfering particle production

**DOI:** 10.1099/jgv.0.002085

**Published:** 2025-04-11

**Authors:** Jacquline Risalvato, James Zengel, Mark Phillips, Ashley Beavis, Ming Luo, Biao He

**Affiliations:** 1 Department of Infectious Diseases, University of Georgia College of Veterinary Medicine, Athens, GA 30601, USA; 2 Department of Chemistry, Georgia State University, Atlanta, GA 30302, USA; 3 Center for Diagnostics and Therapeutics, Georgia State University, Atlanta, GA 30302, USA

**Keywords:** defective interfering particle (DIP), mumps virus, nucleocapsid protein (NP)

## Abstract

Mumps virus (MuV) is a negative-sense, single-stranded RNA virus belonging to the family *Paramyxoviridae*. MuV causes acute infection of the parotid glands, and the infection can result in severe cases of encephalitis, meningitis and deafness in humans. The non-segmented RNA genome of MuV is encapsidated by the nucleocapsid protein (NP), which forms the ribonucleoprotein (RNP) complex that serves as a template for viral RNA synthesis. To make viral genomic RNA accessible to the viral polymerase, a conformational change within NP occurs. Recently, an atomic model of the NP of MuV was developed with cryogenic-electron microscopy (cryo-EM) using PIV5 NP crystal structure as a homology template. To examine NP’s structure and function, we performed mutational analysis of MuV NP at region(s) proposed to play a role in accessing viral RNA. The MuV NP mutants containing G185P, A197Q, Q200R and groups denoted as Top (N63G, P139D, A197Q), Tip (P109E, N121G, A124R) and Bottom (G21S, E29T, P43N, R93Q, R304Q) were first tested in a minigenome system. All mutations resulted in reduced reporter gene activities with Q200R and Bottom having the most severe negative effects. Rescuing of recombinant viruses with these mutations was attempted, and only MuV mutants ‘185 (G185P)’, ‘197 (A197Q)’ and ‘Top (N63G, P139D, A197A)’ were obtained. The ‘Top’ MuV mutant exhibited normal growth kinetics at low multiplicities of infection (MOIs); however, at high MOIs, the virus had reduced peak litres than low MOIs. Further analysis indicates that production of defective interfering particles (DI particles or DIPs) was enhanced by the mutant virus, indicating that this region, a known alpha-helix hinge region, is important for full-length genome replication, suggesting that it plays a role in maintaining stability of viral RNA-dependent RNA-polymerase on RNP template during MuV viral RNA synthesis. Understanding the production of DI particles will lead to a better understanding of MuV pathogenesis, as well as its replication/transcription process.

## Significance

Mumps virus (MuV) is a re-emerging human pathogen. The nucleoprotein (NP) of MuV and all other paramyxoviruses is essential for RNA synthesis and viral replication. Using mutagenesis, we have identified a region within MuV NP that is critical for MuV viral RNA synthesis. These findings will help lead to a better understanding of MuV and paramyxovirus RNA synthesis.

## Introduction

Mumps virus (MuV) is a single-stranded, non-segmented, negative-sense RNA virus that is a member of the *Paramyxoviridae* family and genus *Orthorubalavirus* [1]. This human pathogen can be transmitted through the upper respiratory tract or conjunctivae by droplet transmission among individuals to cause a disease known as mumps [2,3]. Infection typically results in malaise and hallmark swelling of the parotid glands. In severe cases, the infection can result in meningitis, encephalitis and deafness. Additionally, one in four post-pubescent males will develop orchitis, with about 13% of these patients developing subfertility [4,5]. Up until the introduction of mass vaccination of the MMR (measles, mumps and rubella) vaccine in the 1960s, mumps was the leading cause of acquired sensorineural deafness in children [6]. After the introduction of a nation-wide, two-dose, MMR vaccination programme in 1989, mumps was considered nearly eliminated with less than 0.1 mumps cases per 100,000 people by 2001 in the USA [7,8]. However, recent outbreaks in the past decade among younger vaccinated populations indicate the need for the development of new strategies for outbreak control.

Three key proteins are involved in MuV replication: nucleoprotein (NP), phosphoprotein (P) and the large protein (L) [9,10]. Together, L and cofactor P form the viral RNA-dependent RNA-polymerase (vRdRp) for viral RNA replication and transcription. NP forms a helical capsid that supports and protects the RNA genome [11,12]. P is bound to NP by the amino acid terminal (P_N_) and carboxy-terminal domains (P_C_). While the P_N_ plays a role in unravelling the NP so that L may access the RNA genome for replication, P_C_ transports L to the NP-RNA template [13]. The vRdRp is responsible for the transcription of the RNA genome, as well as the 5′ capping and addition of the poly(A) tail on the 3′ end of viral mRNA [9,13]. Together, these three proteins form the vRNP (viral ribonucleoprotein) complex. The vRNP is a well-established feature of paramyxoviruses, with the NP structure among different viral species being relatively conserved [10,14].

The structure of MuV NP has only recently been determined, with atomic modelling achieved using the cryogenic-electron microscopy (cryo-EM) map onto a PIV5 NP crystal structure framework due to PIV5’s NP having approximately 64% sequence homology to MuV NP [15]. This publication has allowed for a more structural analysis and function prediction of MuV NP. For example, amino acid residues 180–202 have been proposed to open and expose the RNA genome for vRdRp access, which is often referred to as the ‘hinge’ region [13,15, 16]. PIV5 NP’s region aligns similarly, however, there are several key amino acid residue differences within the region, primarily at 185, 197 and 200, which are major sites for stability and function within this hinge region. However, functions of these regions have not been investigated experimentally.

Additionally, other functionally conserved regions, such as P–NP binding regions for chaperoning in PIV5 (referred to in our work as the ‘Top’ region based on its location within the NP subunit), L binding stabilization (referred to in our work as the ‘Tip’ region) and structural integrity of NP subunit-to-subunit binding for proper ring formation (referred to in our work as the ‘Bottom’ region) did not have identical nor structurally similar amino acid residues between MuV NP and PIV5 NP [15]. These differences within conserved regions raised many questions regarding whether these regions were truly conserved between the two paramyxoviruses in their function, or if the residue differences between them allowed for different functionalities of NP between the two viruses.

To understand the structure and functions of MuV NP, we developed our own theoretical three-dimensional (3D) model for MuV NP using PIV5 NP crystal structure and MuV NP cryo-EM model. We then mutated residues within MuV NP to corresponding PIV5 NP residues and analysed their impact on MuV replication: mutations were evaluated using a minigenome assay first, and then incorporated into a full-length MuV and analysed.

## Methods

### Mapping of MuV NP onto PIV5 NP

To create a theoretical model of the MuV NP, we utilized the Swiss-Model [12,17], which is a web-based tool for automated comparative modelling of 3D protein structures. The amino acid sequence of the MuV NP was input into Swiss-Model. For the template, we selected the crystal structure of the parainfluenza Virus 5 (PIV5) NP (PDB ID: 4XJN.1.C) [15,18], due to its structural similarity and relevance.

The Swiss-Model server aligned the MuV NP sequence with the PIV5 NP template and generated a theoretical model by mapping the MuV NP amino acid sequence onto the PIV5 NP structure. This process involved several steps, including template identification, target-template alignment, model building and model quality evaluation.

After generating the initial theoretical model, we compared it with the experimentally determined crystal structure of the MuV NP (PDB ID: 7EWQ), which was obtained from the RCSB Protein Data Bank. This comparison allowed us to validate our theoretical model and make any necessary adjustments to improve its accuracy. The final model was refined based on these comparisons to ensure it closely resembled the actual structure of MuV NP.

### Molecular cloning

Plasmids used in these experiments were constructed using standard molecular cloning techniques (details and files available upon request). MuV isolated in Iowa from 2006 (GenBank: JN012242.1) was used as a basis for plasmid sequence [19]. MuV helper plasmids NP, P and L have been previously cloned into the pCAGGS expression vector [20,21]. Firefly luciferase (pFF-Luc) and a MuV minigenome plasmid expressing *Renilla* luciferase flanked by MuV-IA trailer and leader sequences under a T7 promoter (pT7-MG-RLuc) were also previously described [22].

### Cell culture and transfections

Vero and 293T cells were maintained in Dulbecco’s modified Eagle medium (DMEM) with 5% FBS and 1% penicillin–streptomycin (P/S) (Mediatech Inc., Manassas, VA). BSR-T7 cells were maintained in DMEM supplemented with 10% FVBS, 1 % P/S and 10% tryptose phosphate broth (TPB). Cell lines were incubated at 37 °C with 5% CO_2_. Cells were passed at an appropriate dilution 1 day prior to use to obtain 60–90 % confluency upon transfection or infection. JetPRIME transfection reagent (Polyplus Transfection Inc., New York, NY) was used to transfect cells following the manufacturer’s protocols.

### MuV minigenome system and dual-luciferase assay

BSR-T7 cells at 60–80 % confluency in a 24-well plate were transfected with pCAGGS-P (80 ng), pCAGGS-L (500 ng), pT7-MG-RLuc (100 ng), pFF-Luc (1 ng) and various amounts of pCAGGS-NP (wild-type or mutant at 0, 25, 50 or 100 ng) using jetPRIME (Polyplus Transfection, France) according to the manufacturer’s protocol. pCAGGS-GFP (green fluorescent protein) was used to maintain a constant amount of total plasmid transfected per well and served as a positive control for successful transfection. After 48 h, the medium was removed, and 100 µL of passive lysis buffer (Promega, Madison, WI) was added to each well. The plates were then shaken on an orbital shaker for 20 min. 40 µl of lysate was then transferred to a white, flat-bottom, 96-well plate, while the remaining 60 µl of lysate was frozen at −20 °C for analysis via Western blot. A dual-luciferase assay (Promega) was performed according to the manufacturer’s protocol. A GloMax 96-microplate luminometer (Promega) was used to detect luminescence. The ratio of Renilla to firefly luminescence was determined for each well as the ‘relative luciferase activity’, and the average and standard error of triplicates was calculated. For Western blot analysis, mouse monoclonal anti-MuV NP and P antibodies were used together to detect NP and P expression, respectively, as previously described [11].

### Virus rescue

The 293T cells at 60–80 % confluency were transfected in a six-well plate with pCAGGS-NP (200 ng), pCAGGS-P (320 ng), pCAGGS-L (1250 ng), pCAGGS-T7 (200 ng) and full-length MuV genome (2,500 ng) using JetPRIME (Polyplus). After 48 h, the cells were co-cultured with Vero cells at a ratio of 1 : 2 in a 10 cm dish, and supplemented with DMEM with 5% FBS and 1 % P/S. After 24 h, the media was replaced with DMEM with 2% FBS and 1 % P/S. Cells were observed for an additional 2–7 days after co-culture until cytopathic effect (CPE) was observed, at which time single plaques were isolated and expanded in Vero cells in six-well plates to establish passage 1 (P1). Then, P1 titre was determined by plaque assay and passaged again (P2) in T75 flasks at a multiplicity of infection (MOI) of 0.01. Virus was collected after 72 h into aliquots and stored at −80 °C with sucrose phosphate glutamate (SPG). Plaque assays were performed to determine the viral titre. The virus sequence was confirmed by RT-PCR and sequencing.

The viral genome was confirmed via extraction from cell culture supernatants using a QIAamp Viral RNA Mini Kit (Qiagen Inc., Valencia, CA). Reverse transcriptase PCR (RT-PCR) was performed using a OneStep RT-PCR system (LifeTechnologies) and five primer sets that provide full genome coverage. These PCR products were purified using a QIAquick PCR Purification Kit (Qiagen Inc., Valencia, CA) and submitted with sequencing oligomers for Sanger Sequencing.

### Immunoblotting of virus

Viral supernatant was collected and diluted to a titre of 3.0×10^6^ PFU ml^−1^. Virus samples were diluted 3 : 1 with 4× Laemmli Sample Buffer (Bio-Rad Laboratories, Hercules, CA) plus β-mercaptoethanol at 9 : 1, and then heated at 95 °C for 5 min. Samples were loaded into a 4–20 % Mini-PROTEAN^®^ TGX (Bio-Rad Laboratories, Hercules, CA) polyacrylamide gel, and proteins were size-separated by gel electrophoresis. The proteins were then transferred to a polyvinylidene difluoride (PVDF) membrane (GE Healthcare, Piscataway, NJ). The membrane was then incubated with mouse anti-MuV-NP antibody (1 : 2,000 dilution), followed by incubation with Cy3-conjugated goat anti-mouse IgG secondary antibody (1 : 2,500 dilution) (Jackson ImmunoResearch, West Grove, PA), and scanned using a Typhoon 9700 imager (GE Healthcare Life Sciences, Piscataway, NJ).

### Growth curves

Confluent Vero cells in a six-well plate were infected with MuV (wild-type or MuV-NP Y185P, A197Q or Top mutant) at a MOI of 0.01 or 2 in 1 ml of DMEM with 2% FBS and 1 % P/S for 2 h in triplicate. Cells were then washed three times with media, and 2 ml of DMEM with 2% FBS and 1 % P/S was added to the cells. An initial sample was taken immediately after the DMEM was added to the cells and labelled as 0 h post-infection (hpi). For the high MOI of 2, the samples were collected at 0, 12, 24, 36, 48 and 72 hpi. For the low MOI of 0.01, the samples were collected at 0, 24, 48, 72, 96 and 120 hpi. All samples were supplemented with SPG and stored at −80 °C. Viral litres were determined by plaque assays on Vero cells. Each experiment was repeated for confirmation.

### Haemagglutination assay

Whole cockerel erythrocytes were obtained from the Poultry Diagnostic Research Center (Athens, GA, USA) as a gift from Brent Lovern. The red blood cells (RBCs) were centrifuged at 4 °C for 10 min at 150 rcf. After the centrifugation, the serum was removed, retaining the RBC pellet and PBS was added back to the original volume. This washing process was repeated a total of four times to create a 5% stock solution, viable for up to 7 days. The washed RBCs were then diluted to a 0.25% concentration in PBS. In a 96-well V-bottom plate, MuV was diluted with PBS in sequential 1 : 2 dilutions in a total volume of 50 µl per well, to which 50 µl of 0.25% RBCs were added to each well in addition. The plates were incubated at 4 °C for 90 min, and agglutination patterns were observed.

### Viral reduction in infectious yield assay

Vero cells were grown to confluency in 24-well plates in DMEM with 5% FBS and 1 % P/S. MuV-*Renilla* luciferase was prepared at a MOI of 5. MuV or mutant MuV was diluted to MOIs of 2, 0.1, 0.01 or 0.001. The MOI groups were halved, with one-half being treated with UV light (UV Stratalinker 1800) at 10×10^3^ µJ×100 five times. Cells were then infected with MuV-*Renilla* luciferase, and then with either the UV-treated or untreated MuV or mutant MuV at various dilutions in addition. Cells with virus were incubated for 2 h at 37 °C with 5% CO_2_ with a total volume of 250 μl of DMEM with 2% FBS and 1 % P/S and virus. After incubation, cells were washed twice with DMEM with 2% FBS and 1 % P/S, and then covered with 500 μl of media.

At 24 and 48 h, the media was removed, and cells were washed with PBS and then lysed with *Renilla* luciferase Assay Lysis Buffer (Promega) according to the manufacturer’s protocol, with shaking on an orbital shaker for 30 min. The cell lysates were then collected and added to a white, flat-bottom, 96-well plate. The *Renilla* luciferase Assay Protocol (Promega) was then followed according to the manufacturer, and luminescence was measured using the GloMax 96-well plate luminometer (Promega). An average between triplicates was taken for luciferase activity, and the data were normalized to the activity of MuV-*Renilla* luciferase infection alone. Experiments were done in triplicate and repeated twice for consistency.

### RT-PCR of DI particles

Viral RNA was extracted from cell culture supernatants using a QIAamp Viral RNA Mini Kit (Qiagen Inc., Valencia, CA). RT was performed with random hexamers and SuperScript III reverse transcriptase (Life Technologies). The cDNA templates along with four unique leader and trailer specific primers and one control primer pair specific to the F and HN genes of the MuV genome were used to amplify DI particles and fragments of the MuV and mutant genome by PCR. PCR products were examined by electrophoresis on a 1% agarose gel and then gel extracted and column purified using the QIAquick Gel Extraction Kit (Qiagen Inc., Valencia, CA). Primer sets were run in duplicate and experiments were run twice to confirm banding patterns for reproducibility. Primer sets were used on multiple passages to account for passage population differences.

### TOPO cloning and sequencing

Purified RT-PCR products were cloned into the pCR™ 2.1-TOPO® TA vector using the TOPO® TA Cloning Kit (Invitrogen, Carlsbad, CA). Reaction products were then bacterially transformed into TOP10 *Escherichia coli* cells (Invitrogen, Carlsbad, CA). Five bacterial colonies from each cloning experiment were selected and grown overnight in culture at 37 °C, and their plasmid DNA was extracted using the QIAprep Spin Miniprep Kit (QIAgen Inc., Valencia, CA). Primers for the M13F (5′-GTAAAACGACGGCCAG-3′) and M13R (5′-CAGGAAACAGCTATGAC-3′) regions of the pCR™ 2.1-TOPO® TA vector were used for Sanger sequencing by Genewiz (South Plainfield, NJ). Sequences were analysed using Geneious 11.1.5 (Auckland, New Zealand).

### Analysis of chromatogram peak variance

Background peak variance to quantify the heterogeneity of mutant and wild-type virus samples that were Sanger sequenced was calculated using the following formula:


$$100\times \frac{\text{Variant Base Peak Height}}{\text{Primary Base Peak Height}+\text{Variant Base Peak Height}}$$

This formula was adapted from the ThermoFisher Scientific Minor Variant Finder Software v 1.0 User Guide and computational program [23,25].

### Statistical analysis and replication statement

Statistical analysis was performed using Graphpad Prism software version 5.04 for Windows (Graphpad Software, La Jolla, CA). Student’s *t*-test was used to calculate *P* values for the growth curves, and one-way ANOVA was used for comparing groups of the reduction in infectious yield assays. Experiments were run in triplicate, with each experiment run a minimum of two separate times to confirm outcomes.

## Results

### Identification of the residues that may play a role in allowing access to viral RNA

**Fig. 1.**
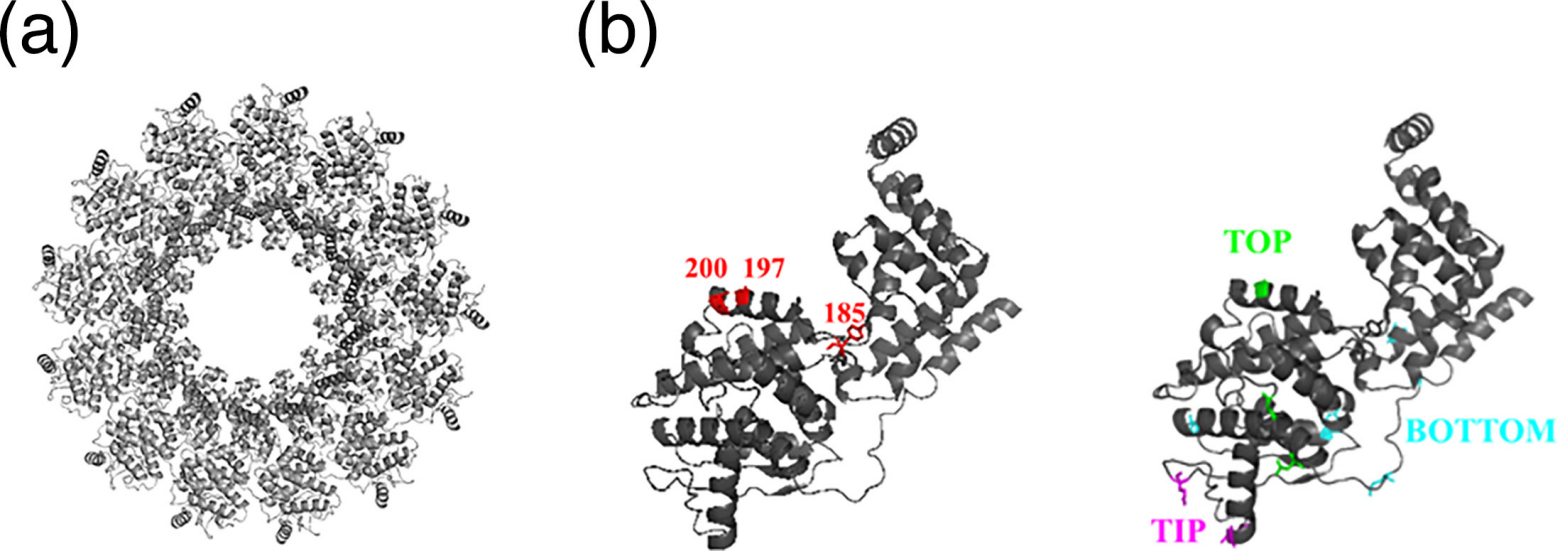
*Model of MuV nucleoprotein*. (**a**) The resulting 3D model of MuV NP built upon a PIV5 NP crystal structure and shifted to MuV EM modelling. The image(s) shown depict the ring structure composed with 13 NP subunits. Red sites are 185 and 200, while green, magenta and cyan residues are ‘Top’, ‘Tip’ and ‘Bottom’ domains, respectively. Panel (**a**) is depicting a full ring structure of Mumps nucleoprotein. 13 NP subunits make up one ring that holds the MuV RNA genome, with stacks of these NP rings forming a helical nucleocapsid. (**b**) Depicts a singular NP subunit. Sites 185, 197 and 200 are marked and highlighted in red, and domains ‘Top’, ‘Tip’ and ‘Bottom’ are denoted in green, magenta and cyan, respectively, within the NP subunit model. Images were designed in PyMOL software.

To determine the key residues that may play a role in allowing access to viral RNA during viral RNA synthesis, we constructed a theoretical 3D model for MuV NP using the known PIV5 NP crystal structure as a template. This model was then used to make structural comparisons to known regions of PIV5 and MuV using crystal structure and cryo-EM studies (respectively) [12,18, 19]. By comparing the cryo-EM model of MuV NP and the PIV5 NP crystal structure, we were able to identify the differences between them [15] (Fig. 1). There were six key individual and regional residue differences between PIV5 and MuV NP within the ‘core’ of MuV, residues 1–379, that are hypothesized to have potential effects on viral RNA synthesis (Table 1). The highly unstable C-terminal tail (residues 402–571) was not included as it has not been shown to be utilized in viral RNA synthesis nor is essential for NP formation *in vitro* for cryo-EM modelling [18,26].

**Table 1.** Construct names and mutations made to the NP protein

Mutant	Site of mutation
185 (Individual)	Y185P
197 (Individual)	A197Q
200 (Individual)	Q200R
Top	N63G/P139D/A197Q
Tip	P109E/N121G/A124R
Bottom	G21S/E29T/P43N/R93Q/R304Q

Three of these differences were singular sites (MuV to PIV5): Y185P, A197Q and Q200R. It is hypothesized that MuV L opens a ‘hinge’ region within NP between amino acid residues 180–202 to expose the RNA genome for vRdRp – but this region is poorly conserved between MuV and PIV5, marking it as a region of interest in this study. Amino acid residues 197 and 200, specifically, are believed to be on the NP helix that must be shifted during a P-initiated conformational change for L to access the RNA genome, whereas 185 is upstream of the hinge region and plays a role in the hinge region’s flexibility [18,19, 27].

The latter three differences were grouped into regions based on their structural location in NP: ‘Top’ (N63G, P139D, A197Q), ‘Tip’ (P109E, N121G, A124R) and ‘Bottom’ (G21S, E29T, P43N, R93Q, R304Q). Many of the amino acid residue differences were within the N-terminal globular domain, with ‘Bottom’ encompassing both the N-arm and C-terminal domains and residing on the non-RNA interacting side of NP – indicating its importance to MuV NP’s structural integrity. ‘Top’ and ‘Tip’ domains are areas hypothesized to interact with the carboxy-terminus of MuV’s phosphoprotein – making this an area of interest to investigate and compare with PIV5’s NP structure, as PIV5’s NP is not documented to interact with the carboxy-terminus of its phosphoprotein [26,28,30]. To assess these differences and how they might affect the structure and function of the RNP, amino acid residues in MuV NP were mutated to those corresponding ones in PIV5.

### Effects of NP mutations in a minigenome system

**Fig. 2.**
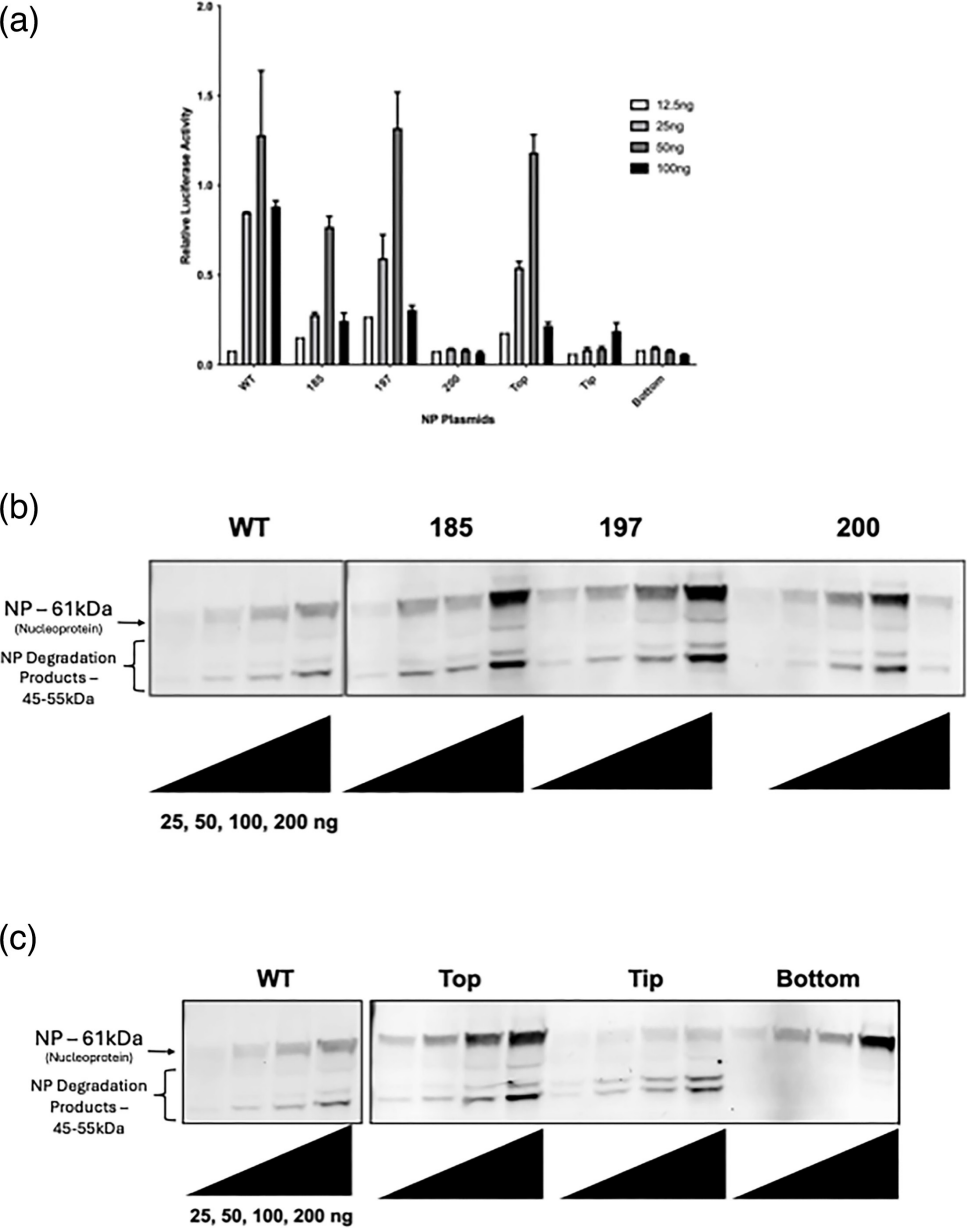
*Effects of mutations in NP in a minigenome system*. (**a**) Relative minigenome activity for NP mutants 185, 197, 200, Top, Tip and Bottom. Western blot evaluating qualitative protein expression levels of NP mutants 185, 197 and 200 (**b**) as well as mutants Top, Tip and Bottom (**c**).

To characterize the effects of mutations, mutations were tested in a minigenome system. Lysates were evaluated for *Renilla* luciferase levels as an indication of replication activity of the mumps viral genome and immunoblotted for confirmation of expression. It was found that mutants 185, 197 and 200 expressed well as determined by immunoblotting, but mutants 185 and 200 had decreased minigenome activity when qualitatively compared with wild-type MuV NP in the minigenome system (Fig. 2a, b). Mutant 197 and ‘Top’, with ‘Top’ including the A197Q mutation, had similar minigenome activity compared with wild-type MuV NP. NP mutants ‘200’, ‘Tip’ and ‘Bottom’ had almost no measurable minigenome activity even though their expressions were detected. Interestingly, ‘Tip’ had increased NP degradation product, whereas ‘Bottom’ had little (Fig. 2c).

### Successful rescue and phenotype determination of MuV mutants

**Fig. 3.**
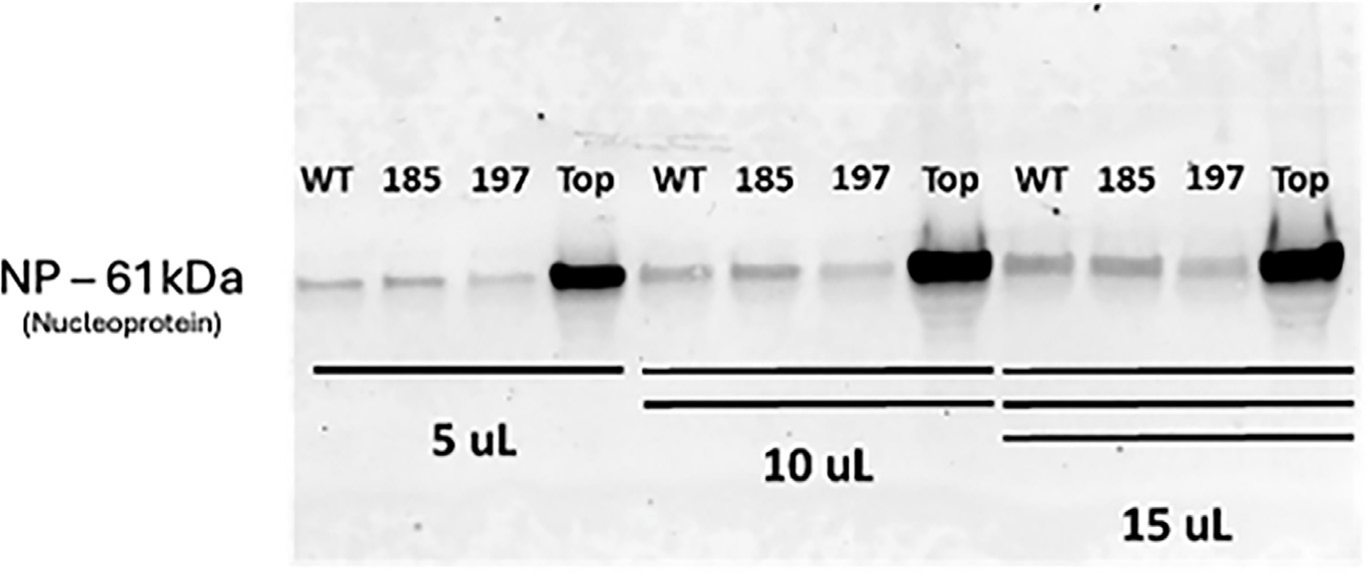
*Detection of NP in media of infected cells*. Viruses MuV-IA (WT), ‘185’, ‘197’ and ‘Top’ were each diluted to 3.0×10^6^ PFU ml^−1^, and either 5, 10, or 15 μl of virus was loaded onto an SDS-PAGE gel. NP was detected using immunoblotting. The passage number for each virus was the same at three passages.

Mutations were incorporated into the full-length MuV genome to evaluate their impact on virus replication. As expected, 197 and ‘Top’ were viable for virus rescue, but unexpectedly 185 was also rescued (Table 2). The sequences of the rescued viruses were confirmed with RT-PCR. The amount of NP in the virus particles was determined via Western blot analysis (Fig. 3) using virus with the same titre (PFU ml^−1^). More nucleoprotein was detected via Western blot per plaque forming unit (PFU) of the ‘Top’ mutant virus compared to wild-type, which may be the result of an altered PFU to virus particle ratio. The ‘Top’ domain contains a mutation at amino acid residue 197, but a mutation at site 197 alone was not sufficient to induce this notable change in increased level of NP in virus (supernatant of infected cells).

**Table 2.** Rescue status of MuV mutants with NP mutations

Plasmid	Alias	Mutation(s)	Successful rescue attempt(s)	Plaques with crystal violet staining	Positive (+) Immuno-staining	Colony purified	Viral Titre
pJR01	MuV NP 185	Y185P	1/1	YES	YES	YES	3.25E+07
pJR02	MuV NP 197	A197Q	1/2	YES	YES	YES	4.70E+07
pJR03	MuV NP 200	Q200R	0/4	NO	NO	NO	_
pJR04	MuV NP TOP	N63G/P139D/A197Q	1/1	YES	YES	YES	2.67E+06
pJR05	MuV NP TIP	P109E/N121G/A124R	0/4	NO	NO	NO	_
pJR06	MuV NP BOTTOM	G21S/E29T/P43N/R93Q/R304Q	0/4	NO	NO	NO	_

**Fig. 4.**
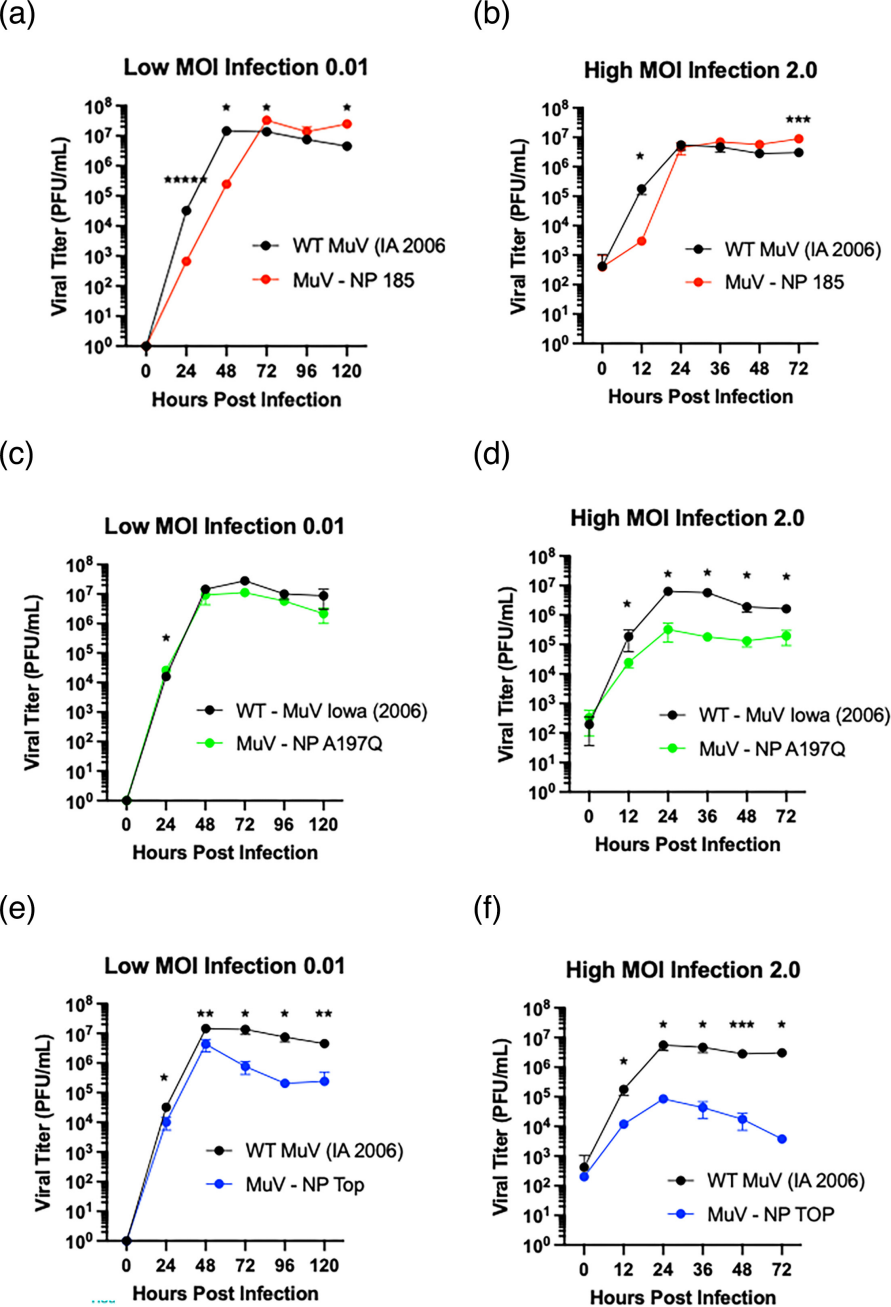
*Growth curve kinetics of MuV mutants compared with wild-type*. Low MOI growth curves (**a, c, e**) for 185, 197 and ‘Top’, respectively, are based on samples taken every 24 hpi for 72 h of a MOI of 0.01 infection of Vero cells. High MOI growth curves (**b, d, f**) for 185, 197 and ‘Top’, respectively, are based on samples taken every 12 h post-infection for 60 h of a MOI of 2 infection of Vero cells. Each MOI infection was verified by back titration. Error bars are standard deviation. A single asterisk ‘*’ denotes statistical significance of *P*<0.05 between groups at the same time point, with ‘**’, ‘***’ and ‘*****’ reflecting a statistically significant difference of *P*<0.01, 0.001 and 0.0001, respectively. Each infection was done in triplicate, and the experiment was repeated a minimum of twice.

Growth kinetics of the viruses were evaluated at low (0.01) and high (2.0) MOIs. Mutant Y185P at both low (Fig. 4a) and high (Fig. 4b) MOI had different growth kinetics from wild-type MuV but reached similar peak litres. Mutant A197Q grew similarly to MuV wild-type when infected at a low MOI (Fig. 4c). However, when infected at a high MOI, A197Q grew one log lower than wild-type by the end of the 72 h collection period (Fig. 4d). Interestingly, ‘Top’ grew approximately one log lower than wild-type by the end of the time collection period when infected at a low MOI (Fig. 4e), and when infected at a high MOI was unable to grow to high titre (Fig. 4f). At 24 hpi, when peak titre for high MOI infections is expected, ‘Top’ grew only two logs higher from its initial titre at hour 0, decreasing thereafter to just above the same titre as the 0 hpi timepoint at the end of the experiment.

### “Top” virus inhibits MuV replication

**Fig. 5.**
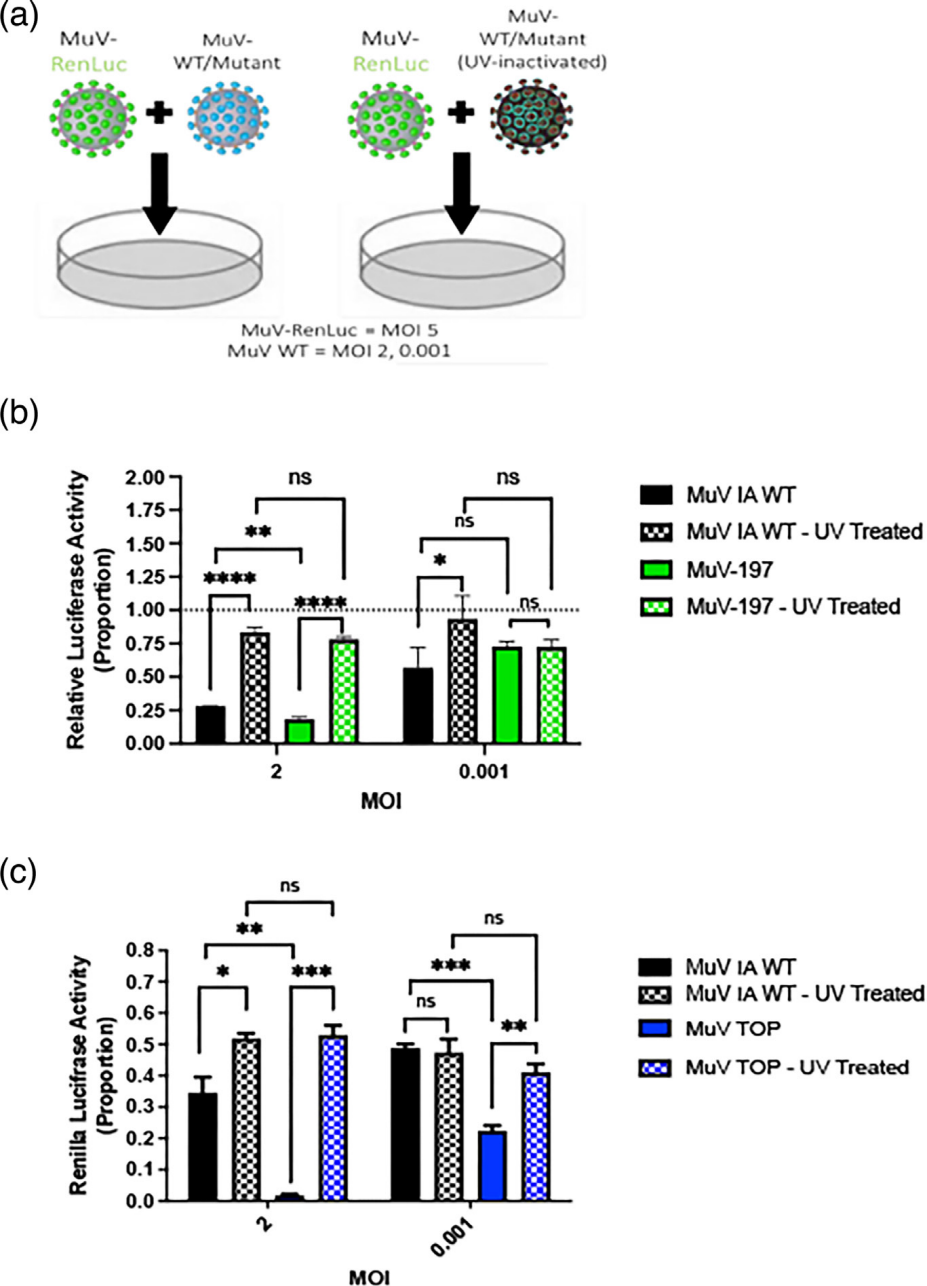
Inhibition of replication assay. A diagram of the experimental set-up and design of the reduction of infectious yield experiments (**a**). The *Renilla* luciferase activity for wild-type and 197 MuV, as well as wild-type and 197 MuV that was UV inactivated, is shown at 48 h (**b**) post-infection at a MOI of 2 and 0.001. The same experiment was done for ‘Top’ MuV, and the Renilla luciferase activity is shown at 48 h (**c**) post-infection. One-way ANOVA was used to compare groups, with error bars representing standard deviations and asterisks denoting *P*<0.05 (*), *P*<0.01 (**), *P*<0.001 (***) and *P*<0.0001 (****). Comparisons with no significant difference are denoted as ‘ns’.

Since high MOI infection resulted in lower virus titre than low MOI infection, we speculate that something in the virus stock might be inhibitory to MuV replication. To investigate whether the ‘Top’ virus (supernatant from ‘Top’ virus infected cells) has an inhibitory effect on MuV replication, an inhibition of replication assay was designed. The experimental design (Fig. 5a) was based on a co-infection of Vero cells with a MuV expressing *Renilla* luciferase (to act as a measurable reporter of MuV replication) and either a wild-type or mutant MuV. The rationale is that if something in the virus stock (supernatant of infected cells) was inhibitory of virus infection, the virus stock should inhibit replication of MuV-*Renilla*. If the component in the virus stock that is inhibitory of MuV-*Renilla* replication is RNA-based, sensitivity to UV cross-link would confirm if the inhibitory effect came from RNA since RNA is sensitive to UV cross-link. The wild-type or mutant MuV was either UV-inactivated or untreated. This UV-treatment should cross-link viral RNA in the media to inactivate the virus and render it ‘sterile’ in culture. If the effect on MuV-*Renilla* luciferase activity remained unchanged after the wild-type or mutant virus was UV-inactivated, then the inhibition of viral replication by ‘Top’ is more likely caused by something in the supernatant that was not RNA-based, i.e. viral RNA. While the MOI of MuV-*Renilla* luciferase will be maintained the same for each co-infection, the wild-type or mutant virus was titrated from high to low MOIs.

For virus A197Q, at 24 hpi (Fig. 5b) the mutant had a significant negative effect on the *Renilla* luciferase activity. At 48 hpi (Fig. 5b), there was a significant difference between the inhibition caused by untreated wild-type MuV and untreated A197Q for the higher of the MOI groups, but not for the low MOI group. In Fig. 5c, at 48 hpi, there was a significant difference in the inhibition of the MuV Renilla reporter by the Top mutant virus at both high and low MOIs. This is further demonstrated by the ‘Top’-UV-treated virus having minimal inhibitory activity compared with wild-type-UV-treated virus, thus confirming that the inhibition in viral growth by ‘Top’ is due to RNA and not a protein-based extracellular component in the supernatant.

### Only infectious virus titre is affected, not binding affinity

To investigate whether the infectious titre of ‘Top’ was accurately representing the presence of total virus particles, a haemagglutination assay was performed. In Table 3, it is shown that compared to wild-type MuV, the amount of haemagglutination activity was much higher than what should be expected of ‘Top’ given its infectious titre. Consistent with the results in Fig. 3, which showed that more nucleoprotein was detected per PFU via immunoblotting compared with wild-type MuV, this result indicates an altered PFU to virus particle ratio. This information leads to the hypothesis that there are virus particles unaccounted for by the plaque titre that are capable of binding to cells but are incapable of plaque formation. A plausible explanation is that the ‘Top’ virus produces a significant amount of DIPs compared with wild-type MuV, and therefore, the growth of plaque-inducing virus is negatively affected.

**Table 3.** Haemagglutination titres compared with infectious virus (PFU) titre of MuV wild-type and mutants

Virus	Passage	PFU ml^−1^	HA Titre	PFU to HA ratio (ml^−1^) (x10^3^)	Normalized to wild-type
MuV WT	5	1.63E+7	128	6.37	1
MuV Ren	5	4.38E+7	256	8.55	1.34
MuV-Y185P	2	3.25E+7	128	12.70	1.99
MuV-A197Q	2	4.70E+7	256	9.18	1.44
MuV-Top	2	3.00E+7	128	1.17	0.18
MuV-Top	3	7.77E+5	256	0.15	0.02

### RT-PCR and sequencing of “Top” confirms the presence of defective interfering particles

DI genomes are generated by most negative-stranded RNA viruses during viral replication. These genomes are truncated forms of the original genome and usually retain elements from its full-length parent genome: a suitable initiation site on the 3′ end and its complement on the 5′ end, the terminal sequence(s) recognized by the vRdRp, and sequences for packaging and encapsulation into the NP [28,30]. Based on these parameters, primers were designed to capture potential DI genomes.

**Fig. 6.**
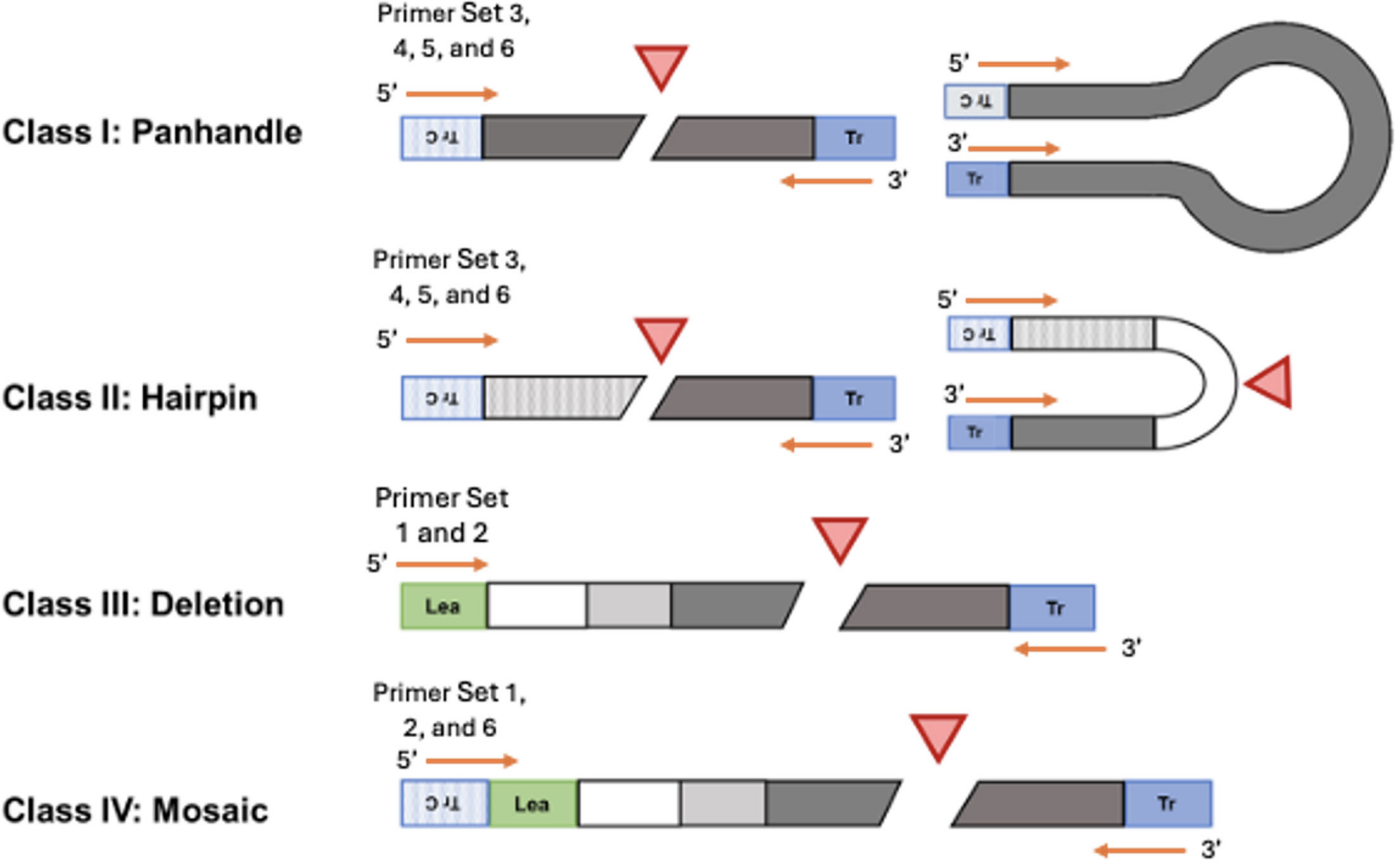
*Diagram of types of defective interfering particle genomes*. The diagram shows four main categories of DI particle genomes: Class I and Class II are referred to often as copy- and snap-backs, respectively, and share similar inherent properties in that the leader/trailer sequences are complementary, representing the skipping of the polymerase between positive and negative RNA strands. Class III is a common type of DI particle, where the polymerase skips large sections of the genome but reattaches on the same strand and direction, causing minor to major deletions in the final replicated genome. The last class, mosaic, represents a combination of the classes within the same DI particle and does not occur often. Primer sets from Table 4 are included for reference.

When considering DI genome classifications, there are four main types: Class I which is ‘panhandle’ or ‘copy-back’, Class II which is ‘Hairpin’ or ‘snapback’, Class III which is an internal deletion and Class IV which is mosaic – a combination of deletions and copy-back DI genome types. These genomes are depicted in Fig. 6 and the primer sets used to capture the DI genomes in Table 4. The most common type of DI genome is Class III, or an internal deletion genome, which would be captured by primer sets 1 and 2. Class I includes ‘copy-back’ or panhandle DI genomes, which arise when the polymerase carrying an incomplete strand ‘switches back’ to transcribe the 5′ end, forming a classic panhandle shape in the RNA sequence. Class II includes hairpin and ‘snapback’ DI genomes, which are less complex in their secondary structure than copy-backs; in this case, the polymerase will transcribe part of one strand and then use it as a template, forming a hairpin structure [31,32]. It was hypothesized that copy-back and hairpin DI genomes were intended to be captured by primer pairs 3, 4, 5 and 6; primer set 6 would use the leader sequence and its complement to capture Class I and II DI genomes, while sets 3, 4 and 5, as single primers, would help discern copy-backs from hairpins. Typically, if there is a complex secondary structure, such as a panhandle, the polymerase during PCR will either skip over the structure and make shortened cDNA segments with internal deletions or will generate truncated products. Hairpins typically have fewer inner deletions with shorter segments. These structures are detailed further in Fig. 6. The mosaic form was not targeted specifically in this work but could be captured by primer sets 1, 2 and 6 as they target leader and trailer sequences.

**Table 4.** DIP primer pairs list for RT-PCR of MuVs. Seven primer pairs were used in all, with three being single primers and pair “7” being a positive control for the F and HN gene region of the MuV genome. Primer descriptions and sequences are included

Primer set	Forward primer	Reverse primer	Description	RT-PCR bands present in MuV-WT pass 2?	RT-PCR bands present in MuV-Top pass 2?	RT-PCR bands present in MuV-Top pass 3?
1	JR236F:5′-ACC AAG GGG AAA ATG AAG ATG GGA TAT TGG TAG AAC AAA TAG TGT AAG AAA CAG T-3′	JR234R:5′-ACC AAG GGG AGA AAG TAA AAT CAA T-3′	Entire leader sequence with trailer sequence	No	Yes (2)	Yes (1)
2	JR232F:5′-ACC AAG GGG AAA ATG AAG ATG GGA TAT T-3′	JR234R:5′-ACC AAG GGG AGA AAG TAA AAT CAA T-3′	First half of leader sequence with trailer sequence	Yes (4)	Yes (3)	Yes (5)
3	JR236F:5′-ACC AAG GGG AAA ATG AAG ATG GGA TAT TGG TAG AAC AAA TAG TGT AAG AAA CAG T-3′	na	Entire leader sequence only	No	Yes (1)	No
4	JR232F:5′-ACC AAG GGG AAA ATG AAG ATG GGA TAT T-3′	na	First half of the leader sequence only	Yes (3)	Yes (3)	Yes (6)
5	na	JR234R:5′-ACC AAG GGG AGA AAG TAA AAT CAA T-3′	Trailer sequence only	No	Yes (1)	No
6	JR232F:5′-ACC AAG GGG AAA ATG AAG ATG GGA TAT T-3′	JR238R:5′-AAT ATC CCA TCT TCA TTT TCC CCT TGG T-3′	First half of the leader sequence with its reverse complementary primer	Yes (1)	Yes (smear)	No
7	MuV_4939 F5′-ACA AAT GCA CGC GCA ATA GC-3′	MuV_8550R: 5′-AAC TGA CAG GCA AGC CAA AC-3′	3.6 kb region within F and HN genes	Yes (1)	Yes (1)	Yes (1)

**Fig. 7.**
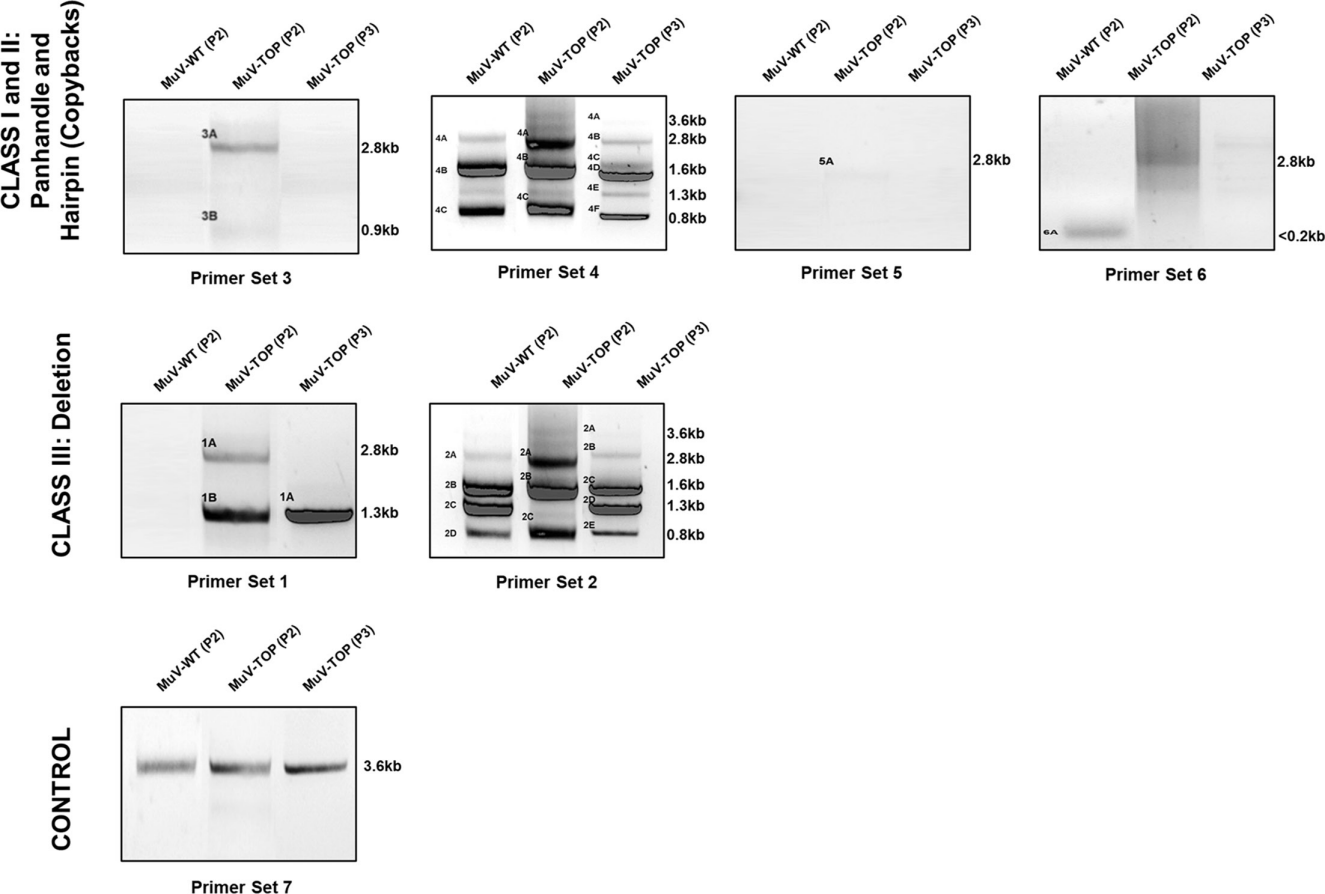
*RT-PCR of MuV using primers designed for detecting DIPs*. Included are images of the gel electrophoresis of the RT-PCR products of extracted viral RNA from passage 2 of MuV wild-type and ‘Top’ mutant passages 2 and 3. Primer sets are organized by their ‘class’ of DI particles and labelled at the bottom of the image. Bands are labelled starting with their primer set number and then with a letter of the alphabet in order of size (largest to smallest). Bands are also labelled with relative sizes to the right of each gel image.

To definitively confirm the presence of an increased number of DI particles and to characterize them, wild-type and ‘Top’ virus were serial passaged at low MOI (0.01) for four passages, as beyond passage 4 ‘Top’ exhibited instability with reversions of mutated sites back to wild-type variants. RNA was extracted from these viruses at each passage, and reverse transcribed with random hexamers, and then PCR with leader and trailer primers (Table 4) was performed. In Fig. 7, the wild-type MuV displayed PCR bands for Primer Sets 2, 4 and 6, which included leader and trailer, leader only and leader with its reverse complement, respectively. This was consistently observed throughout passages 2, 3 and 4 (data not shown), establishing a qualitative ‘baseline’ of DI particle genomes that may appear naturally in the wild-type MuV population.

‘Top’ (passage 2) presented bands for the same primer groups as wild-type MuV (2, 4, 6); however, it also had bands for groups 1, 3 and 5, which included the primers for the full leader and trailer, the full leader alone and the trailer alone, respectively (Fig. 7). Interestingly, Primer Set 6 (leader with reverse complement) for ‘Top’ showed a large DNA smear. This was likely due to when amplifying reverse-transcribed RNA from ‘Top’ using complement leader primers, there was increased DNA of various lengths, indicating a high likelihood of the presence of copy-back genomes that could not be well-transcribed due to various secondary structures [31]. ‘Top’ passage 3 appeared to have ‘lost’ some of the PCR bands that hallmarked ‘Top’ passage 2 (Fig. 7), indicating that with serial passages, the viral population has a shift back to the wild-type genotype and thus becomes heterogeneous.

**Fig. 8.**
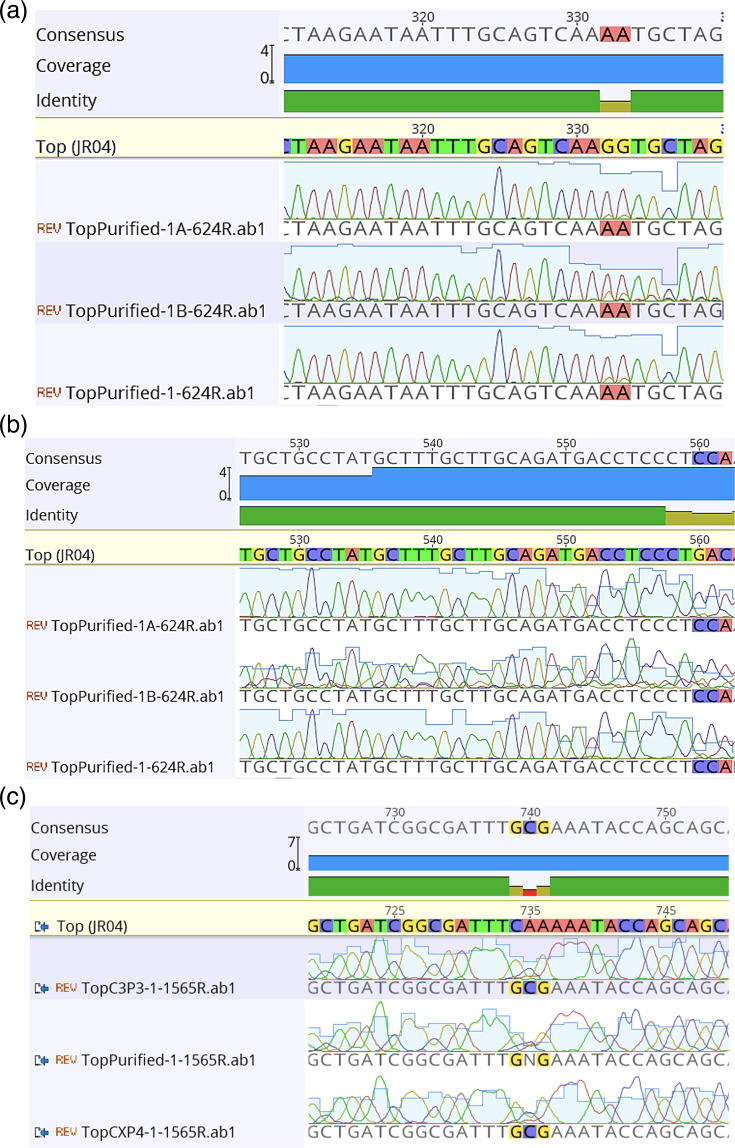
*Sanger sequencing results from TOPO cloning of excised RT-PCR DIP gel electrophoresis products*. As seen in Fig. 7, ‘Top’s’ population becomes more heterogeneous and reverts to a predominantly wild-type genotype. Through TOPO cloning of excised gel fragments of the RT-PCR products, Sanger sequencing was able to be performed on these unknown band sequences captured by the DI primer sets. This qualitatively observed variability from the gel electrophoresis results is further supported by the variance of peak height and quality surrounding the base pairs, which should be mutated in passage 4 of the ‘Top’ virus, for amino acid sites 63 (**a**), 139 (**b**) and 197 (**c**). A collection of sequencing samples is shown and is aligned to the sequence for ‘Top’ virus.

To determine DI sequences, the bands from wild-type MuV and ‘Top’ passages 2 and 3 were cloned and then sequenced. In Fig. 8, Sanger sequencing analysis for bands consistent between wild-type and ‘Top’ passage 2 showed an increased variety of deletion defects in the sequences, while sequences of bands unique to ‘Top’ passage 2 revealed the presence of copy-back sequences. ‘Top’s’ heterogeneity increased as the passage number increased, as can be shown when looking at amino acid residues 63 (Fig. 8a), 139 (Fig. 8b) and 197 (Fig. 8c) and calculations on chromatogram data (data not shown).

Taken together, the sequencing and RT-PCR data suggest three main aspects regarding ‘Top’ and its production of DI particles: (1) ‘Top’ produces a greater number of DI particles compared to wild-type MuV, (2) ‘Top’ produces a greater variety of DI particles, particularly copy-back, compared with wild-type MuV, and (3) as ‘Top’ is passaged, the DI particle genotype becomes more representative of wild-type’s DI particle profile in its increase in deletion and decrease in copy-back DI genomes.

## Discussion

Here, we have shown that by mutating MuV NP amino acid residues to corresponding PIV5 residues critical to NP stability and vRdRp interactions in a crystal structure, MuV NP function is affected. Three of the mutations (200, ‘Tip’ and ‘Bottom’) proved to be detrimental to replication and virus viability. The mutation at 185 showed comparable growth kinetics to wild-type MuV, with growth being significantly delayed initially but recovering to higher litres than wild-type. As this is the mutation that was in the flexible hinge over the RNA binding groove, it is possible that the initial delay is due to slowed viral replication kinetics. Mutant 197, with its mutation within a loop important for forming the RNA binding groove and a potential site for P_C_ binding, showed decreased viral growth at high MOI infection. Interestingly, ‘Top’, a MuV mutant where amino acid residues at 63, 139 and 197 were altered, showed similar peak litres at low MOI infection and displayed defective growth kinetics when infected at a high MOI (Fig. 4). When investigating the possibility of ‘Top’ alone being the culprit of these inhibitory effects seen in MuV growth, it was determined that not only at high MOI but also at low MOI, ‘Top’ was able to inhibit viral replication of MuV-*Renilla* luciferase. This observation was further confirmed by the fact that when ‘Top’ is UV-inactivated, these inhibitory effects were no longer observed (Fig. 5). Furthermore, ‘Top’ displayed binding affinities by HN to sialic acid in litres comparable to wild-type virus, indicating that there were virus particles in the supernatant of ‘Top’ because they were non-infectious, and thus they were being unaccounted for by plaque assay (Table 3) . All these results have led to the hypothesis that the ‘Top’ mutant produces more DI particles.

DI particles were first discovered in 1954 by Preben von Magnus while working with influenza virus [32,33]. Non-influenza RNA viruses, specifically paramyxoviruses, produce DI segments and particles of potential inhibitory qualities [34,37]. RNA viruses can produce DI genomes, resulting in the activation of the innate immune system [38,39]. There is a great interest in these DI particles because of their potential as an adjuvant in stimulating both mouse and human dendritic cells [40,42].

Data in this work suggest that the ‘Top’ domain of the nucleoprotein plays a role in its ability to keep the polymerase complex to the RNA template during elongation. When this site was mutated, the change resulted in an unstable viral RdRp complex: the complex switches template to produce a shortened genome, i.e. DI particles. It is possible that this ‘Top’ domain has decreased affinity to interact with vRdRp, resulting in the phenotype since this region is proposed to interact with vRdRp. This potential deficiency in interaction needs to be further investigated. Since it is known that the ‘Top’ domain falls within the N-terminal region of MuV NP that is critical for the formation of the RNA groove, interactions between the ‘Top’ domain and vRdRp are required to expose the template RNA and maintain the stable structure of the translocating replication complex, and thus proper genome replication by L is altered in the mutants, resulting in increased DI particle production.

With interest in using DI particles as adjuvants, it will be of great significance to investigate whether similar mutations in other paramyxovirus NP proteins will have similar phenotypes. This work will aid in developing further understanding of paramyxovirus replication and potential use of DIPs.

## Abbreviations

cryo-EM: cryogenic-electron microscopy
DIP: defective interfering particle
DMEM: Dulbecco’s modified Eagle medium
hpi: hours post-infection
L: large protein
MMR: measles, mumps and rubella
MOI: multiplicity of infection
MuV: mumps virus
NP: nucleocapsid protein
P: phosphoprotein
PFU: plaque forming unit
P/S: penicillin–streptomycin
PVDF: polyvinylidene difluoride
RBC: red blood cell
RNP: ribonucleoprotein
RT-PCR: reverse transcriptase PCR
SPG: sucrose phosphate glutamate
TPB: tryptose phosphate broth
vRdRp: viral RNA-dependent RNA-polymerase
vRNP: viral ribonucleoprotein
